# Human Tumor-Infiltrating Dendritic Cells: From In Situ Visualization to High-Dimensional Analyses

**DOI:** 10.3390/cancers11081082

**Published:** 2019-07-30

**Authors:** Margaux Hubert, Elisa Gobbini, Nathalie Bendriss-Vermare, Christophe Caux, Jenny Valladeau-Guilemond

**Affiliations:** Cancer Research Center Lyon, UMR INSERM 1052 CNRS 5286, Centre Léon Bérard, 28 rue Laennec, 69373 Lyon, France

**Keywords:** dendritic cells, cytometry, RNA-seq, single cell RNA-seq, immune signatures, prognosis

## Abstract

The interaction between tumor cells and the immune system is considered to be a dynamic process. Dendritic cells (DCs) play a pivotal role in anti-tumor immunity owing to their outstanding T cell activation ability. Their functions and activities are broad ranged, triggering different mechanisms and responses to the DC subset. Several studies identified in situ human tumor-infiltrating DCs by immunostaining using a limited number of markers. However, considering the heterogeneity of DC subsets, the identification of each subtype present in the immune infiltrate is essential. To achieve this, studies initially relied on flow cytometry analyses to provide a precise characterization of tumor-associated DC subsets based on a combination of multiple markers. The concomitant development of advanced technologies, such as mass cytometry or complete transcriptome sequencing of a cell population or at a single cell level, has provided further details on previously identified populations, has unveiled previously unknown populations, and has finally led to the standardization of the DCs classification across tissues and species. Here, we review the evolution of tumor-associated DC description, from in situ visualization to their characterization with high-dimensional technologies, and the clinical use of these findings specifically focusing on the prognostic impact of DCs in cancers.

## 1. Introduction

Although they represent only a very small proportion of leukocytes, dendritic cells (DCs) are central to the immune system [1]. The interaction between tumor cells and the immune system is considered to be as a dynamic process [2,3,4] and, owing to their sentinel cell properties, DCs are at the heart of early stage of tumor immune surveillance [3]. DCs are at the interface between innate and adaptive immunity through their involvement in naïve T cell priming based on their ability to sense danger signals, migrate to secondary lymphoid organs, and present antigen (Ag). Since the original characterization of DCs in humans, technological advances have enabled the identification of several distinct DC populations [5] according to their origin, localization, phenotype and functions. Each DC subset has several specificities, allowing the immune system to recognize a multitude of danger signals inducing an adapted response via the synthesis of multiple cytokines and the shaping of T lymphocytes differentiation. As a result of the variety of markers, tissues and inflammatory contexts analyzed, the classification of DCs has until recently lacked homogeneity. Several methodologies are currently used to define the different sub-populations [6,7]. They include surface phenotyping, generally performed by flow cytometry [8,9], ontogeny analysis to identify their progenitors and the transcription factors involved in their development [10,11,12,13], as well as their functional characterization [5]. However, a relatively recent consensus has emerged on a universal and simplified classification of DC subsets both in mice and in humans [11,14]. Furthermore, high- dimensional molecular analyses have arisen, providing a comprehensive as well as an extensively detailed portrait of the tumor microenvironment, and further improving our knowledge of DC subpopulations [15,16,17]. These different approaches are complementary and provide a description of DCs which is constantly evolving and sometimes controversial. We discuss herein the strengths and weaknesses of each approach in this field, focusing on new high-dimensional technologies and future strategies.

## 2. DC Subpopulations and Their Functional Specificities

**Figure 1:**
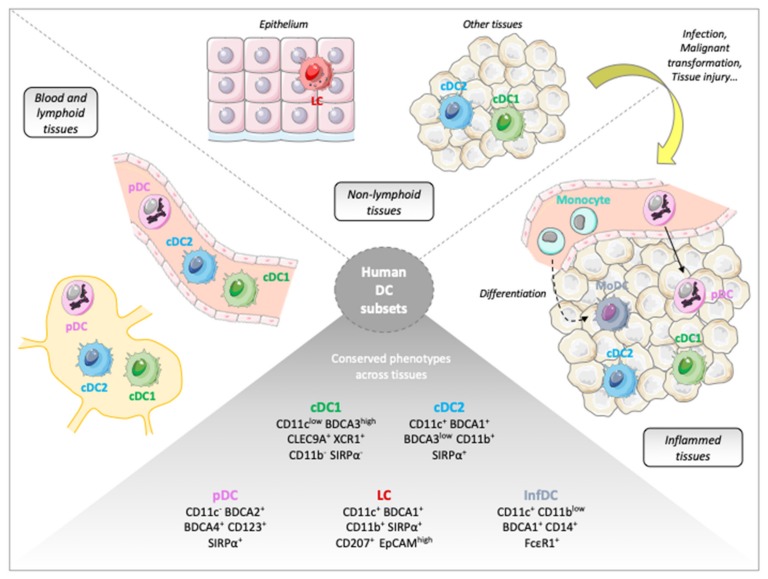
Human Dendritic Cells subsets. DC: Dendritic Cells; LC: langerhans cells; cDC1: conventional dendritic cell type 1; cDC2: conventional dendritic cell type 2; MoDC: monocytes-derived dendritic cells; pDC: plasmacytoid dendritic cells; InfDC: inflammatory dendritic cells.

Both human (Figure 1) and murine DC subsets exhibit a comparable organization. Although resident and migratory DCs display differences, the classification separating plasmacytoid and myeloid/conventional lineages is applicable to blood and lymphoid tissue-associated DCs [8,9]. The particular DC population present in the stratified epithelia are called Langerhans cells (LCs). In addition, inflammation induces the differentiation of monocytes into dendritic cells (MoDCs), also called inflammatory DCs (infDCs), and the tissue infiltration by circulating pDCs (Figure 1).

### 2.1. Plasmacytoid Dendritic Cells (pDCs) and Anti-Tumor Immunity

Even though pDCs contribute to the activation of T cell-mediated adaptive immune responses via Ag presentation [18,19,20], their major known function resides in their capacity to secrete important quantity of type I interferon (IFN-I) [21,22]. The recognition of nucleic acids by the toll-like receptors 7 and 9 (TLRs) expressed in their endosomal compartments induces their key production of IFN-α, particularly in the context of viral infection [23,24,25,26]. Moreover, IFN-I are crucial cytokines involved in the anti-tumor immunity [27,28]. Thus, the potential role of IFN-I in controlling tumor growth was first evidenced through the use of neutralizing antibodies leading to the favored development of tumors in mice [29]. Mice deficient in IFN-β genes or IFN-I receptors were subsequently also shown to be more sensitive to tumor induction and aggressivity [30,31,32,33]. Consistently, it was shown that the negative regulation of the expression of the human IFN-I receptor is associated with a reduced survival in patients with colorectal carcinoma, whereas a strong IFN response signature is associated with a good outcome [34]. In addition, the anti-tumor role of IFN-I appears to be a consequence of pleiotropic immunomodulatory functions in mice, rather than through a direct effect on tumor cells [29,30,31]. Indeed, in several murine cancer models, the IFN-I family is involved in the control of metastatic dissemination by activating the killing capacity of NK cells and cytotoxic CD8^+^ T cells against tumor cells [35,36,37]. IFN-I is also implicated in the initiation of anti-tumor cytotoxic T lymphocyte (CTL) responses by promoting survival and cross-presentation of DCs [32,35,38,39] as well as their differentiation, maturation and cytokine production [40,41,42]. Finally, IFN-I can inhibit regulatory T cells (Tregs) [43,44,45,46] and myeloid derived suppressor cells (MDSCs) [47,48], considered to be immunosuppressive cells contributing to tumor development. However, the preferential production of IFN-α by pDCs and their role in tumor immune surveillance remains to be demonstrated.

### 2.2. Conventional Dendritic Cells Subsets and Anti-Tumor Immunity 

Conventional DCs (cDCs), also known as classical or myeloid DCs (mDCs) differ from pDCs, particularly in the expression of the CD11c membrane marker (alpha X integrin), as well as a higher expression of class II MHC molecules (HLA-DR) at steady-state. This family of cells can be subdivided into two distinct subpopulations of cDCs, according to their phenotype and functional specialization, historically called BDCA3/CD141^high^ and BDCA1/CD1c^+^ DCs [8,9]. Analyses of their transcriptomic profile have demonstrated their similarities with murine DCs and identified equivalent mouse populations. In order to standardize and uniformize the nomenclature used to cite BDCA3/CD141^high^ and BDCA1/CD1c^+^ populations, they are now commonly referred to cDC1 and cDC2 respectively [11]. In mice, cDC2 correspond to the resident and migratory CD11b^+^ cDCs, whereas cDC1 can be further divided into two phenotypically different subsets: the lymphoid tissue-resident CD8α^+^ cDCs and the migratory CD103^+^ CD11b^−^ cDCs.

#### 2.2.1. cDC1 

Cytokine secretion by cDC1 reflects an important role of these cells in the induction of Th1 lymphocytes responses. Actually, human and murine cDC1 represent the main producers of IL-12p70 in response to TLR stimulation [49,50]. After TLR3 engagement, cDC1 produce inflammatory cytokines and chemokines, such as IL-6, IL-8, TNF-α, CXCL10 [49,51,52] as well as the relatively newly discovered type III IFN/IFN-λ [52,53,54]. The phenotype and functions of cDC1 also indicate close interactions between this specific DC subset and cytotoxic lymphocytes. They express NECL2/CADM1 [55,56] and XCR1 [51,55,57,58], involved in the interaction of cDC1 with NK and CD8^+^ T cells [56,57,58,59]. Moreover, XCR1 allows the recruitment of cDC1 through the recognition of the XCL1 chemokine produced by these activated cytotoxic lymphocytes [60,61,62]. Many studies conducted in mouse models have shown the superiority of cDC1 to cross-present soluble or cellular Ag on class I MHC (MHC-I) molecules in order to activate effective CTL responses [63,64,65,66], in particular in viral contexts [67,68,69,70]. This superior Ag cross-presentation ability of cDC1 compared to other DC subsets was also highlighted in human [49,50,51,60,71]. Nevertheless, the capacity of human cDC1 to cross-present Ag remains to be confirmed in the context of tumors. Furthermore, the cDC1-specific expression of CLEC9A/CD370 could explain the functional superiority of cDC1 to cross-present Ag associated with necrotic cells [49,50,72], as this C-type lectin enables the endocytosis of necrotic material [73,74,75]. Our team contributed to highlight the existence of an important cross-talk between cytotoxic lymphocytes and cDC1 by demonstrating that TNF-α and IFN-γ production by NK cells potentialize the Ag cross-presentation by cDC1 [76]. 

Given their ability to activate cytotoxic immune responses, the role of cDC1 in anti-tumor immunity has been extensively investigated in mice. *Batf3*^−/−^ mice, a model of cDC1 deficiency, allows to demonstrate their crucial involvement in the initiation of CTL responses leading to tumor rejection [32,35,66]. Several studies demonstrated the superiority of murine tumor associated cDC1 (TA-cDC1) to engulf tumor material [77,78] and to migrate to the secondary lymph nodes [78,79,80,81], where they can efficiently activate T cells by Ag presentation [77]. Other interesting data suggest a crucial role for cDC1 in the response to immunotherapies. Indeed, cDC1 are essential for the response to anti-immune checkpoints, such as anti-PD-L1, anti-CTLA4 and anti-CD137 [81,82,83], and to adoptive transfer of anti-tumor T cells [77,84], since treatment efficacy is lost in cDC1 deficient mice. 

#### 2.2.2. cDC2

The major population of human myeloid DCs in blood, lymphoid organs and non-lymphoid tissues are cDC2. This population can sense many danger signals owing to their expression of a wide range of pattern recognition receptors (PRRs) shared with monocytes (TLR1, 2, 4, 5, 6 and 8) [85] or multiple lectins (CLEC4A, CLEC7A, CLEC6A, CLEC10A and CLEC12A) [5]. After engagement of these receptors, human cDC2 can produce a large variety of cytokines such as TNF-α, IL-1β, IL-6 and IL-8 [86]. Likewise, they secrete large amounts of IL-12p70 [86,87,88] and appear to be the main producers of IL-10 and IL-23 [51,87,88,89,90]. In addition to the production of cytokines, it has reported that cDC2 can induce the differentiation of naïve T cells into Th1, Th2 and Th17 [87,88,91], demonstrating their ability to induce a broad spectrum of immune responses. Finally, cDC2 also engage in Ag cross-presentation, effectively activating CD8^+^ T cells [72,86,92,93]. Unlike cDC1 that are critical for directing the CD8^+^ anti-tumor immunity, cDC2 preferentially initiate CD4^+^ T responses in diverse contexts [80,94,95,96]. Of note, Laoui et al. suggested their potent induction of Th17 polarization of CD4^+^ T cells in mice [80]. Interestingly, a recent study also highlighted the suppressive effects of regulatory T cells on cDC2 function and the importance of the cDC2/Treg balance on the quality of T cell responses and the patient’s prognosis [96]. 

The absence of cDC2 in *Irf4^−/−^* mice led to the conclusion that *Irf4* is required in the development of this subset [97,98]. Thus, functions of murine cDC2 are mostly investigated in *Irf4* deficient mice. However, a fraction of cDC2 is still present in this mouse model with a defective migration due to an impaired induction of CCR7 [99]. Hence, it appears that *Irf4* is not strictly required for the development of the cDC2 population and that no perfect model of mice with a specific cDC2 deficiency exists [5], not facilitating their functional characterization.

### 2.3. Other DC Populations

In addition to pDCs and cDCs, other populations of DCs are present in peripheral tissues (Figure 1). Langerhans cells (LCs) are located in epithelial tissues such as the epidermis. They specifically express the C-type lectin CD207/Langerin [100]. In the standard commonly used classification [11], LCs are categorized in the monocytic lineage due to their origin and the factors influencing their differentiation common to the macrophage lineage [101,102]. However, if we focused on the LCs functions rather than on their ontogeny, these cells can clearly be considered as DCs. Indeed, studies in humans have shown that LCs from lymph nodes are able to induce the differentiation of naive CD4^+^ T cells into Th2 helpers or to efficiently activate CD8^+^ T cells [103,104]. Furthermore, although LCs are completely different from cDC1, they share the strong expression of some common genes involved in Ag cross-presentation [104], a function LCs appear to be efficient in [104,105,106]. 

In inflammatory contexts, monocytes are able to differentiate into several cell types including inflammatory DCs, also called monocyte-derived DCs [107,108]. Although the ability of monocytes to differentiate into infDCs has long been demonstrated in vitro in the presence of GM-CSF and IL-4 [109], evidence of this differentiation in vivo has been long-awaited. The existence of human inflammatory myeloid cells was reported in various pathological contexts such as eczema and psoriasis [110,111]. Several groups also demonstrated the ability of monocytes to differentiate into infDCs at the tumor site [112,113], as well as the existence of systemic cancer-induced factors driving this differentiation in patients’ blood [114]. Although infDCs seem to be derived from monocytes, their expression of several markers such as CD1a, BDCA1 or FcεR1, as well as their functional properties, can enable authors to classify them as DCs. This cell type is for example capable of responding to danger signals by expressing costimulatory molecules and by inducing T cell responses [114]. 

## 3. Detection of DCs in Solid Tumors

### 3.1. In situ Visualization of Tumor-Associated DCs 

DCs were identified in human tumors mainly by in situ immunohistochemistry (IHC). The first studies were based on in situ S-100 protein staining to detect myeloid cells [115]. Based on this marker, tumor-associated DCs (TA-DCs) were described in gastric, breast, ovarian, colon, lung, kidney, bladder, and head and neck cancers. However, since some DCs do not express it and, though other myeloid cells such as CD163^+^ macrophages do [115], this marker does not allow the distinction between all myeloid subpopulations and these results should therefore be considered caution. Subsequently, the use of complementary markers such as DC-LAMP, CD83 or CD86 has facilitated the study of the state of the maturation of TA-DC (Table 1). It seems that TA-DCs with an intra-tumor localization are generally immature, unlike TA-DCs located in the peritumoral zone or in the invasive margins. In addition, mature TA-DCs appear to interact closely with T cells in peritumoral areas [116].

As described above, DC subsets have many particularities, rendering the identification of different subtypes within the immune infiltrate essential. This has been achieved via the use of antibodies targeting specific markers of each population, such as anti-CD123 or anti-BDCA2/CD302 antibodies, to demonstrate the presence of pDCs in multiple tumors (Table 1), particularly in breast and ovarian cancers [44,122,131]. However, among tumor-infiltrating immune cells, CD123 expression is not exclusively restricted to pDCs. In addition, the BDCA2 marker is negatively regulated during the pDCs maturation, thus challenging the visualization of mature pDCs. The double in situ CD123 and BDCA2 or CD123 and BDCA4/CD304 staining would therefore provide a more reliable identification of pDCs in situ. The presence of LCs has also been demonstrated with the specific CD207 marker (Table 1) in melanoma, colorectal and breast tumors [116,122]. Moreover, the recent work of Salmon et al. has also demonstrated the presence of cDCs in primary melanoma [81] (Table 1). However, the results of this study may be questionable. Actually, in this study cDC1 are defined by the CD11c^+^ BDCA3^+^ phenotype while the BDCA3 molecule can be expressed by many other cell types, such as cDC2 also defined in this study by the BDCA1^+^ CD20^−^ phenotype. Finally, CD14^+^ BDCA1^+^ cells, thought to be infDCs, were visualized in skin and colon lesions of metastatic melanoma [114].

### 3.2. Identification of TA-DC Subsets by Flow Cytometry

To overcome the limited number of markers visualized by in situ immunohistochemistry analysis, flow cytometry panels have been developed to achieve the simultaneous identification of several DC populations (Table 2). However, this technique requires the dissociation of tissues in order to obtain a cell suspension, impeding the ability to obtain the precise localization of stained cells. Our studies by Sisirak et al. and Labidi-Galy et al. have shown the presence of pDCs (CD11c^−^ CD123^+^ BDCA2^+^) and cDCs (CD11c^+^) in breast and ovarian tumors respectively [44,142]. In breast cancer tissues, we demonstrated the increase in pDC frequency in aggressive tumors, such as triple negative breast cancer (TNBC) or with a high mitotic index [44]. The relationship between the aggressiveness of tumors and their high infiltration by pDCs was also reported in ovarian cancer. Indeed, the pDC proportion increase greatly in tumors of patients during disease progression compared to patients in complete remission [142]. 

More recently, the addition of complementary markers has enabled the identification of several subtypes of cDCs by flow cytometry. For instance, BDCA3^+^ cDC1 and BDCA1^+^ cDC2 have been identified in the CD16^−^ HLA-DR^+^ CD11c^+^ CD14^−^ cell population infiltrating metastatic melanomas [77], but also in lung, colorectal and breast tumors [77,80,143]. The proportion of cDC subsets appears to be equivalent in colorectal cancer and in melanoma, while a higher percentage of cDC1 than cDC2 is found in non-small cell lung carcinomas (NSCLC) [80]. Although these studies represent considerable progress by demonstrating the diversity of DC populations associated with human tumors, the absence of antibodies targeting specific markers of cDCs is regrettable. The staining of CLEC9A and/or XCR1 would validate the nature of the identified cDC1. Finally, the presence of CD16^−^ BDCA1^+^ CD14^+^ infDCs has been shown in lung and colorectal cancers [80], as well as in melanoma lesions-draining lymph nodes and in head and neck squamous cell carcinoma tumors [96], and in two types of inflammatory samples: synovial fluid from arthritic joints and ascites from patients with breast or ovarian cancer [144].

## 4. High-Dimensional Technologies Applied to DC Subset Analyses

### 4.1. Limitations of Previous Techniques and Emergence of High-Dimensional Technologies

Though advances have been made, the accurate identification of DC sub-populations remains difficult with the classical technologies in order to phenotypically and functionally characterize those cells. The tissue environment and the state of maturation of DCs can also significantly influence their phenotype and functional properties, resulting in the long-standing existence of various nomenclatures between species or tissues to define the same cell population. The concomitant development of high-dimensional technologies, such as mass cytometry (CyTOF) or complete transcriptome sequencing (RNA-seq), has provided an extensive characterization of previously identified populations [149]. These techniques have led to the discovery of new subsets, but also highlighted similarities between populations of different species leading to a standardized DCs classification across tissues and species [11]. However, many studies presenting RNA-seq analyses of DCs were based on flow cytometry sorted populations. This technique requires a great strictness in the selection of DC markers, as contamination by other cells can dramatically bias the resulting transcriptomic profile [150]. Conversely, new technical advances made it possible to develop RNA-seq analysis at the single cell level (scRNA-seq). This technology avoids the preliminary sorting step and therefore any potential contaminations, resulting in an unbiased identification of cells a posteriori according to their transcriptomic profile. With both mass cytometry and scRNA-seq, multiparametric analysis and hierarchical clustering provide a reliable ontogenic read-out revealing differences due more to a different state of the same cell population than to an actually existing new cell subset. This type of information could be very useful in the standardization of the DC classification, simplifying the large amount of data available in the literature on this issue.

### 4.2. Inter-Tissue and Inter-Species Similarities

RNA-seq studies highlighted homologies between DC subpopulations in different tissues [13,14,71,151,152] and between different species [51,55,71,153,154,155,156]. Recently, by transcriptomic analysis of DC subpopulations sorted from various human lymphohematopoietic organs, Heidkamp et al. demonstrated that these subtypes are firstly defined by their ontogeny rather by transcriptional programs activated within the different tissues [152] (Table 3). However, these authors also demonstrated that the transcriptomes of DC subpopulations present in non-hematopoietic tissues (skin and lung) are clearly distinct from similar subsets isolated from lymphoid organs, revealing that peripheral tissue-derived signals have a strong influence on their transcriptional regulation [152]. Interestingly, they proposed a combination of biomarkers for the identification of the three major DC subpopulations in a consistent manner across either the lymphohematopoietic or other tissues analyzed (CD123 and CD303 for pDCs, BDCA1 and CLEC10A for cDC2, BDCA3 and CLEC9A for cDC1 in combination with HLA-DR, CD11c, CD11b, CD14 and a lineage cocktail to exclude T, B and NK cells). However, we cannot extend these results to all non-lymphohematopoietic tissues other than those explored in this study. Alcàntara-Hernàndez et al., also recently showed that human skin cDC1 and cDC2 had a different phenotype from their blood and lymphoid organ counterparts according to CyTOF analysis [149].

The alignment of DC subtypes between mice and humans is another crucial issue in order to correlate mouse in vivo experiments with human studies. Therefore, the demonstration of homologies between human and murine DC subtypes [51,55,156,157] represented a major advance in the field and facilitated the analysis of the functional specialization of DC populations. In addition, the development of mass cytometry represented a considerable step forward, allowing both the unsupervised identification of different DC populations and their multiparametric phenotypic characterization. CyTOF technology has recently been used to identify and compare different DC subpopulations across tissues and species (humans, mice, macaques) under normal or inflammatory conditions [15]. The authors thus proposed a universal strategy for DC identification regardless of species and tissues from which they originated. Interestingly, with a scRNA-seq analysis of myeloid cells, Binnewies et al. suggested that human cDC2 subpopulations identified in tumor-draining lymph nodes or in the tumor microenvironment appeared to be very similar to mouse cDC2 [96].

RNA-seq was also useful in highlighting the similarities or differences that may exist between DCs derived from in vitro progenitors and those found in vivo. For example, a study in mice showed that DCs derived from monocytes (in the presence of GM-CSF) had a transcriptomic profile closer to monocytes and macrophages than lymph node-isolated DCs [55]. In humans, CD11b^+^ DCs obtained in vitro from CD34^+^ hematopoietic stem cells (HSCs) appeared to be equivalent to infDCs rather than cDC2. However, the same ex vivo differentiation model enabled the generation of a large number of cDC1 with a transcriptomic profile close to those isolated from blood of healthy donors [52]. A CD34^+^ HSC differentiation protocol is currently available to obtain both plasmacytoid and myeloid lineages (including cDC1 and cDC2 subtypes), with transcriptomes strictly close to those of the same types of cells isolated from blood [161].

### 4.3. Redefining DC Classification

As explained above, scRNA-seq analysis provides an unbiased identification of different cell populations. While recent studies are able to identify all the major immune cell populations present in blood or in tissues, it is very difficult to detect minor cell types such as dendritic cells [162]. The transcriptional profiling at a single cell level is a useful approach to provide a snapshot of the cellular composition of a given tissue, but it may be necessary to enrich rare cell types in the sample prior to scRNA-seq. This strategy was recently and concomitantly adopted by two groups. The work by Villani et al. based on the scRNA-seq analysis of the lineage^−^ HLA-DR^+^ cells of healthy donors’ blood revealed the existence of two new cell populations [17] (Table 3). The first is composed of very early progenitor cells characterized by the expression of CD34^+^ CD100^+^ CD123^low^ markers, with a high differentiation capacity into cDC1 and cDC2. The second population is characterized by the expression of the AXL and SIGLEC6 markers, and is called AS-DC (also called DC5 in their classification) [17]. This population expresses molecules common to pDCs, including some traditionally used for their identification and was therefore isolated with pDCs in cell sorting processes until now, thus likely biasing the functional analysis of these cells. Indeed, unlike pDCs, AS-DCs do not produce IFN-I after engagement of TLR9 but synthesize IL-8 or IL-12p70. Moreover, in addition to their effective T cell activation, this population can differentiate into cDC2, suggesting that they may represent a pre-cDC population [17]. By focusing their scRNA-seq analysis on the HLA-DR^+^ CD135^+^ fraction of PBMCs, consisting both of DC subsets and their progenitors, See et al. also identified this AXL^+^ SIGLEC6^+^ DCs as a population of early precursors with the ability to differentiate into cDCs [16] (Table 3). However, it remains unclear whether DC subsets identified by scRNA-seq conserved their phenotypic characteristics at a protein level. To address this question, Alcantara-Hernandez et al. characterized human DC subsets from blood and lymphoid organs through comprehensive protein profiling at a single-cell resolution by CyTOF [149]. Authors identified a cluster of cells localized near pDCs in the ViSNE plot. This cluster included at least three cell subsets including the AXL^+^ ones. In the same paper, authors performed an inter-individual comparison of blood and skin DCs. cDC1 and pDC phenotypes were consistent across donors for both tissues considered, while cDC2 displayed a relevant inter-individual heterogeneity that could be explained by the differential expression of molecules involved in Ag uptake, presentation, T cell co-stimulation and inhibition [149].

### 4.4. High-Dimensional Technologies Adapted to DC Subset Analysis in the Context of Tumors

The CyTOF technology led to the identification of pDCs (CD123^+^) and cDCs (CD11c^+^) in human clear renal cell carcinoma [158]. However, since the objective of the authors was to perform a large immune profiling of kidney tumors, few markers were dedicated to the analysis of DC subsets. In their study, Lavin et al. also used CyTOF in conjunction with a single cell RNA sequencing to characterize the immune infiltrate of non-small cell lung tumors (NSCLC), in comparison with healthy pulmonary tissues collected at a distance from the tumor and with patient PBMCs [159]. This method confirmed the presence of cDC1 and cDC2 in lung tumors as previously demonstrated by FACS [80]. Moreover, this analysis highlighted the under-representation of cDCs in lung tissues (healthy and tumor) compared to PBMCs, as well as the increased frequency of cDC1 in tumor areas compared to healthy lung tissue [159]. Furthermore, given the expression of monocytic markers by some CD1c^+^ DCs, the author suggested the existence of tumor-associated infDCs [159]. Even though if the phenotype of infDCs is very close to monocytes and cDC2, the transcriptome analysis of BDCA1^+^ CD14^+^ cells isolated from PBMCs of healthy donors revealed a distinct population with a unique gene expression profile [114] (Table 3). Other groups performed transcriptomic analyses of TA-DC subsets. Michea et al. identified and analyzed the transcriptome of human breast tumor-associated pDCs, cDC2 and a cDC1-enriched population [143] (Table 3). They were not able to identify a clear cDC1 subset due to the lack of a specific marker for this rare population. They also analyzed non-malignant tissue adjacent to tumor tissue to determine how tumor-infiltrating DCs adapt to their microenvironment. pDCs harbor the most plastic transcriptome, which is highly impacted by the tumor-derived signals [143]. Moreover, the subtype of breast tumor seems to influence the transcriptomic profile of DC subsets. Indeed, the authors interestingly showed that TNBCs promote a shared signature in DC populations, including the upregulation of the IFN pathway [143]. Finally, Zilionis et al. very recently used scRNA-seq to map immune cell gene expression in human and mouse lung cancer [160] (Table 3). The unbiased comparison revealed a broad conservation of major gene expression programs between these two species. They also showed that the human lung tumor-infiltrating DC compartment contains four distinct subsets present in all patients, albeit in variable proportions [160]. Of interest, they identified a particular DC subset characterized by an activated phenotype (DC-LAMP^+^) and the absence of subset-specific markers. Even though this “DC3” population expresses *BATF3*, a transcription factor involved in the cDC1 differentiation, it completely lacks the expression of *CLEC9A* or *XCR1* [160]. Nevertheless, depending on the scRNA-seq technology used, the expression of some genes may be false negative results and have to be further analyzed.

### 4.5. Transcriptomic Signatures and Prognostic Impact of DCs on Cancer Patients

Some immune populations present in tumors may be indicative of an immune response in patients but can also predict their survival or response to certain treatments such as immunotherapies [163,164]. The presence of DCs in tumors has thus been extensively evaluated in particular to assess their prognostic impact. Similarly to TA-DC identification, initial studies used immunohistochemistry in situ methods to evaluate the prognostic impact of DCs. A majority of these studies demonstrated that a strong expression of the S-100 marker is associated with improved patient survival in many cancers. However, as discussed above, this strategy for identifying TA-DCs lacks specificity. Moreover, the prognostic impact of DC depends on various factors including their state of maturation, as demonstrated by DC-LAMP expression (Table 4). Owing to the improved ability to detect pDCs in large cohorts of patients, several studies have demonstrated the association between the high level of tumor infiltration by this population and a poor patient prognosis, especially in breast and ovarian tumors [44,122] as well as in melanoma [146].

Several strategies are now used to design gene signatures to identify a specific cell type. In effect, transcriptome analysis of a particular subtype of DC results in the identification of the most abundantly expressed transcripts in this population. The comparison across different DC sub-populations has led to the identification of specific or enriched genes in a given population compared to others. These signatures can then be used to establish a score of infiltration by different types of DCs into a specific tissue or immune context. The availability of public transcriptomic databases has facilitated the analysis of DC infiltration in many human tumors has been assessed using these signatures. Broz et al. were the first to use transcriptomic data generated from murine DCs to extract a cDC1 signature, composed of human ortholog genes, as well as an "other myeloid cells" signature [77]. The expression of the genes included in these signatures was then evaluated in the public transcriptome database TCGA (The Cancer Genome Atlas). A high “signature cDC1/signature other myeloid cells” ratio was thus significantly associated with an improved survival in 12 cancer types [77]. The same ratio has also recently been associated with a good prognosis using another breast tumor transcriptomics database (Metabric), again supporting the positive impact of cDC1 tumor infiltration on patient survival [143]. However, the use of human orthologous of murine genes that were identified by RNA-seq analysis is a questionable strategy. Indeed, the study did not demonstrate that human DCs have the same gene signature. Barry et al. nevertheless re-used this cDC1 signature very recently demonstrating the association between a strong cDC1 signature enrichment and improved survival in patients with metastatic melanoma [182]. They also showed a correlation between the tumor infiltrating lymphocyte (TIL) quantitation score and the cDC1 signature [182], a reminiscent result of the correlation between cDC1 and CD8^+^ T cell infiltration scores already reported in melanoma [84]. In order to generate a cDC1 signature, Böttcher et al. used a cDC1 signature composed of 4 genes, the expression of which is restricted to this population: *CLEC9A*, *XCR1*, *CLNK* and *BATF3*. This signature score was then analyzed in the TCGA database, showing that a strong cDC1 tumor infiltration is associated with an improved survival in patients with head and neck, breast or lung cancer and metastatic melanoma [183]. Interestingly, the recent work of Michea et al. provided for the first time a comparison of the prognostic impact across DC subpopulations [143]. Using the Metabric breast tumor database, they demonstrated the association between an important tumor enrichment with the signatures of three DC subsets (pDCs, cDC2 and cDC1-enriched cells) and an improved survival. In TNBC specifically, only the cDC1-enriched cell signature appeared to be associated with a favorable prognosis. However, these results should be interpreted with caution due to the absence of specific markers used in cDC1 and cDC2 sorting [143]. Finally, Zilionis et al. recently used their scRNA-seq data of lung adenocarcinoma-associated DCs to establish gene signatures of immune cells and demonstrated the favorable prognostic impact of DC subsets on patient survival [160,184].

## 5. Perspectives for of TA-DC High-Dimensional Analyses

High-dimensional technologies allow the generation of large amounts of data leading to the precise characterization of immune cells. The identification of novel subsets is a major consequence of transcriptional characterization of DCs. But this should be validated by functional analysis to confirm the robustness of hypotheses emerging from transcriptomic data. The discovery of AXL^+^ cDC precursors among pDCs is an interesting example [16,17]. Even though the authors seriously question the capacity of T cell activation through Ag presentation that was allocated to pDCs by previous studies [20], expanded functional analyses will be needed to confirm that the contamination by cDC precursors was at the origin of the demonstration of T cell activation by pDCs.

An important future objective will be the comparison of TA-DC subsets with DCs from the blood or healthy tissues of patients to identify markers or pathways that could be targeted in tumors. Actually, many studies have highlighted the alteration of DCs by the tumor microenvironment, such as the complete inhibition of IFN-α production by TA-pDCs [44,126,142,185,186,187]. The better characterization of these alterations will be crucial to identify immune evading mechanisms used by tumor cells and to develop new therapeutic strategies to restore the activity of DCs, as central anti-tumor immunity orchestrators. Moreover, even though RNA sequencing engenders many interesting hypotheses, results will have to be confirmed at a protein and functional level. Several studies have performed proteomic descriptions of DC subsets [188,189,190], but the complete proteome analysis of tumor-infiltrating DCs will be a major challenge in this field of research. Indeed, it will be useful to determine the global alterations of DC subpopulations at translational and post-translational level induced by the tumor microenvironment. Proteomics and phospho-proteomics analyses have been performed on infDCs to evaluate their modifications in response to TLR ligands during their maturation induced by pathogens [191,192]. Such analyses on TA-DCs will be challenging, mostly owing to their scarcity in tissues.

The use of transcriptomic signatures is currently used to determine the prognostic impact of immune cells on cancer, especially DCs [77,143,160,182,183]. The next step will probably be the transfer of such analyses to tumors from patients treated by novel and promising immunotherapies to determine the predictive impact of TA-DC subsets. The recent work of Barry et al. demonstrated in two clinical studies of anti-PD-1 treated metastatic melanoma patients that the proportion of TA-cDC1 determined by flow cytometry is significantly higher in responders compared to non-responders [182]. These data do not demonstrate that human cDC1s are required for the response to immune checkpoint blockade as in mice [79,83,84,85,86], but confirms the fascination for this population and their implications for the development of new immunotherapies. Furthermore, transcriptomic analyses of TA-DCs in treated patients will be needed to evaluate the impact of such immunomodulatory treatments. Predictive biomarkers specifically describe the expected likelihood of cancer patients responding to a given therapy, but only few approved tissue-based predictive biomarkers are currently available and even fewer are used in the clinical practice. Since biomarkers used in proteomics, genomics, epigenomics and transcriptomics are all good candidates to predict response to cancer treatments, high-dimensional technologies could then be useful in clarifying this really intricate field. Gene expression data on mRNA extracted from formalin-fixed paraffin-embedded tumor samples have been used to explore the tumor-associated immune microenvironment. For instance, several enriched immune cells or cytokine signatures were shown to be associated with a longer response and survival in immune checkpoint inhibitor-treated patients [193,194,195], but their positive predictive value was not sufficient powered. However, samples used for such analyses encompass complex and heterogeneous mixtures of immune and tumor cells possibly affecting the real predictive value of biomarkers, and the scRNA-seq technology may be informative mainly for rare cell subpopulations such as certain DC subsets. Deciphering the predictive role of each immune subpopulation would allow us to pool data in order to define a multiparametric score taking into account different tumor infiltration patterns associated with different outcomes.

Finally, in cancer patients, immune cell landscape can be affected by the site of biological sampling adding an additional degree of complexity to the well-known tumor heterogeneity. Thus, exploring immune cell subpopulations infiltrating the tumor requires a comprehensive understanding of complex cellular phenotypes and their interrelationships in the spatial context of the tissue microenvironment. Flow cytometry or transcriptomic analyses listed in this review completely abrogate the tissue integrity preventing the localization of DCs or other immune cells in tumors. However, standard laboratory methods such as in situ chromogenic or fluorescent immune-staining only favor the testing of one or few protein expressions [158,196]. The emergence of imaging mass cytometry (IMC, CyTOF coupled with a sophisticated imaging system as Hyperion or MIBI-TOF) has resolved overcoming this issue, offering advantages of large protein phenotyping coupled with the tissue distribution of samples [197,198]. For example, Keren et al. clearly underlined the immune infiltrate heterogeneity by analyzing a cohort of human TNBCs using multiplexed ion beam imaging (MIBI) [199]. As expected, they found many differences in both the variety and composition of the immune infiltrate. Furthermore, the expression of immune regulatory proteins (such as PD-1, PD-L1, IDO or LAG3) by distinct cell types is correlated with the spatial architecture of the tissue. When examining clinical data, compartmentalized organization was associated with an increased patient survival regardless of the tumor-infiltrating lymphocytes score [199]. These results link molecular expression profiles and histological features of tumors, highlighting the multiple layer of information that can be obtained by coupling high-dimensional technologies with in situ visualization. Furthermore, another group demonstrated the feasibility of the simultaneous detection of proteins and transcripts using IMC. They developed an approach combining the modification of the RNAscope-based mRNA in situ hybridization protocol [200] (for 4 genes) with multiple protein staining (17 antibodies) in 70 samples from breast cancer patients [201]. The authors thus demonstrated the existence of clusters of CXCL10-producing cells and their correlation with the presence of T cells in the tumor microenvironment [201]. Even though no data are currently available, the spatial identification of different DC subpopulations identified according to their specific phenotypic and functional biomarkers by mass cytometry would certainly provide comprehensive information on a multiplicity of in situ protein and mRNA expressions on a single slide of rare and precious samples. The assessment of tissues and tumors at a cellular resolution, while preserving the information of tissue architecture and cellular morphology, will probably improve the deciphering of cellular interactions and help to uncover new biomarkers.

## Figures and Tables

**Table 1:** In situ visualization of tumor-associated DCs.

Histology	Biomarkers	DC Subset	Localization	Maturation State	Reference
Breast cancer	CD1a, CD207, CD83	DC, LC	Intratumoral	Immature	[116]
	DC-LAMP, CD11c	cDC	Peritumoral	Mature	
	CD1a	LC	Intratumoral	NA	[117]
	CD1a	LC	Intratumoral	NA	[118]
	CD1a, CMRF-44, CMFR-56, CD83	cDC, LC	Intra and peritumoral	Immature	[119]
	CD1a, CD83	DC, LC	Intratumoral	Mature	[120]
	CD1a	LC	Intratumoral	NA	[121]
	CD1a, CD207	LC	Intratumoral	Immature	[122]
	DC-LAMP	DC	Peritumoral	Mature	
	CD123	pDC	Intratumoral	Immature	
	BDCA2	pDC	Intratumoral	NA	[44]
	DC-LAMP	DC	Intratumoral	Mature	[123]
Colorectal cancer	CD1a	LC	Intratumoral	Immature	[124]
	CD83	cDC	Peritumoral	Mature	
	CD1a	LC	Peritumoral	Immature	[125]
	DC-LAMP	DC	Intratumoral	Mature	
	CD1a, CD207, CD123, DC-LAMP	DC, LC, pDC	Intratumoral	NA or mature	[115]
Lung cancer	CD11c, BDCA2, CD83, Lin-	cDC, pDC	Intratumoral	Immature	[126]
	DC-LAMP	DC	Tertiary Lymphoid Structure	Mature	[127]
Melanoma	CD1a, CD207, DC-SIGN, CD206	cDC, LC	NA	NA	[128]
	BDCA2	pDC	NA	NA	
	CD207, CD1a	LC	NA	NA	[129]
	CD1a, DC-LAMP	DC, LC	Intratumoral	Immature	[130]
	CD1a, DC-LAMP	DC, LC	Peritumoral	Mature	
	CD11c, BDCA3	cDC1	NA	NA	[81]
	BDCA1	cDC2	NA	NA	
Ovarian cancer	BDCA2	pDC	NA	NA	[131]
Kidney cancer	CD80, CD83, CD86, HLA-DR, CMH-I, CD54	cDC	NA	Mature	[132]
	CD1a, CD80, CD86, CD83, CMRF-44	cDC, LC	Intratumoral	Immature	[133]
	CD1a, CD40, CD80, CD83, CD86, HLA-DR	cDC, LC	Intratumoral	Mature and Immature	[134]
	CD1a	LC	Intratumoral	Immature	[135]
	CD83	DC	Peritumoral	Mature	
Head and Neck cancer	CD1a, HLA-DR	LC	Intratumoral	Mature	[136]
		LC	Peritumoral	Immature	
	CD1a	LC	Intratumoral	Immature	[137]
		LC	Peritumoral	Immature	
	BDCA2, CD123, HLA-DR	pDC	Intratumoral	Immature	[138]
	CD1a	LC	Intratumoral	Immature	[139]
	DC-LAMP	DC	Peritumoral	Mature	
Bladder cancer	CD83	DC	Intratumoral	Mature	[140]
Gastric cancer	DC-LAMP	DC	Intra and peritumoral	Mature	[141]

DC: Dendritic Cells; LC: langerhans cells; cDC: conventional dendritic cells; cDC1: conventional dendritic cell type 1; cDC2: conventional dendritic cell type 2; pDC: plasmacytoid dendritic cells; NA: not available

**Table 2:** Identification of tumor-associated DC subsets by flow cytometry.

Histology	Markers	DC Subset	Maturation State	Reference
**Breast cancer**	Lin^−^ CD4^+^ CD11c^−^ CD123^+^ BDCA2^+^	pDC	Mature	[44]
	Lin^−^ CD4^+^ CD11c^+^ BDCA1^+^	cDC2	NA	
**Colorectal cancer**	HLA-DR^+^ CD11c^+^ IRF8^+^	cDC1	NA	[80]
	HLA-DR^+^ CD11c^+^ BDCA1^+^	cDC2	NA	
	HLA-DR^+^ CD11c^+^ CD14^+^	MoDC	NA	
**Lung cancer**	CD1c^+^	cDC2/LC	NA	[145]
	CD123^+^ BDCA2^+^	pDC	NA	
	HLA-DR^+^ CD11c^+^ IRF8^+^	cDC1	NA	[80]
	HLA-DR^+^ CD11c^+^ BDCA1^+^	cDC2	NA	
	HLA-DR^+^ CD11c^+^ CD14^+^	MoDC	NA	
**Melanoma**	HLA-DR^+^ BDCA2^+^	pDC	NA	[146]
	HLA-DR^+^ CD11c^+^ BDCA3^+^	cDC1	NA	[77]
	HLA-DR^+^ CD11c^+^ BDCA1^+^	cDC2	NA	
**Ovarian cancer**	Lin^−^ CD4^+^ CD11c^−^	pDC	Immature	[147]
	HLA-DR^+^ CD4^+^ CD123^+^ CD11c^−^	pDC	Immature	[148]
	Lin- CD4^+^ CD11c^−^ CD123^+^ BDCA2^+^	pDC	Mature	[142]
	Lin^−^ CD4^+^ CD11c^+^	cDC	NA	

DC: Dendritic Cells; LC: langerhans cells; cDC1: conventional dendritic cell type 1; cDC2: conventional dendritic cell type 2; pDC: plasmacytoid dendritic cells; MoDC: monocytes-derived dendritic cells; mDC: myeloid dendritic cells; NA: not available.

**Table 3:** Analysis of human DC subsets by high-throughput technologies.

Tissues	Technologies	DC Subsets	Reference
Spleen, Liver, Lung	CyTOF	cDC1, cDC2 and pDCs	[15]
Blood, Skin, Spleen, Tonsil	CyTOF	cDC1, cDC2, LC (not in blood), pDCs and Axl^+^ DCs (not in skin)	[149]
Clear renal cell carcinoma	CyTOF	DCs, pDCs	[158]
Lung adenocarcinoma, normal lung and blood	CyTOF & scRNA-seq	cDC1, cDC2 and pDCs	[159]
Blood, Skin	Microarray analysis of FACS-sorted DCs	cDC1, cDC2, pDCs (blood only), BDCA1^+^ BDCA3^+^ DCs (skin only)	[51]
Gut	Microarray analysis of FACS-sorted DCs	CD103^+^ SIRP-α^+^ DCs, CD103^−^ SIRP-α^+^ DCs, CD103^+^ SIRP-α^−^ DCs	[13]
Blood, Spleen and Tonsil	Microarray analysis of FACS-sorted DCs	cDC1, cDC2 and pDCs	[152]
Blood	RNA-seq	cDC2 and MoDCs	[114]
Breast carcinoma	RNA-seq	cDC1-enriched cells, cDC2, pDCs and MoDCs	[143]
Blood	scRNA-seq of FACS-sorted DCs	cDC1, 2 clusters of cDC2, pDC and Ax^l+^ DCs	[17]
Blood	scRNA-seq of FACS-sorted HLA-DR+ CD135+ cells	cDC1, 2 clusters of cDC2, pDC and Axl^+^ DCs	[16,96]
Melanoma-draining lymph nodes	scRNA-seq of HLA-DR+ cells	cDC1, 3 clusters of cDC2 and mature DCs	[96]
Lung adenocarcinoma	scRNA-seq	cDC1, cDC2, pDCs and mature DCs	[160]

CyTOF: mass cytometry; RNAseq: complete transcriptome sequencing; scRNA-seq: single-cell transcriptome sequencing; FACS: fluorescence-activated cell sorting; DCs: Dendritic Cells; LC: langerhans cells; cDC1: conventional dendritic cell type 1; cDC2: conventional dendritic cell type 2; pDC: plasmacytoid dendritic cells; MoDC: monocytes-derived dendritic cells; mDC: myeloid dendritic cells.

**Table 4:** Prognostic impact of TA-DCs (by in situ or flow cytometry assays.

Marker/Population	Prognostic Impact	Histology	Reference
**CD1a**	**Positive**	Breast	[117]
			[120]
			[121]
		Head and Neck	[165]
			[166]
		Lung	[167]
		Melanoma	[130]
	**Negative**	Osteosarcoma	[168]
		Biliar tracts	[169]
		Colon	[125]
	None	Breast	[122]
		Kidney	[134]
			[135]
			[137]
**DC-LAMP**	**Positive**	Melanoma	[130]
			[129]
		Lung	[170]
			[171]
			[127]
		Ovary	[172]
		Breast	[123]
	**Negative**	Colon	[125]
		Gastric	[173]
	None	Melanoma	[174]
		Breast	[122]
**CD83**	**Positive**	Breast	[120]
		Lung	[175]
		Colon	[176]
		Kidney	[177]
	**Negative**	Kidney	[135]
		Kidney	[134]
			[178]
**cDC2 (BDCA1)**	**Negative**	Lung	[145]
**pDC (BDCA2, CD123)**	**Positive**	Breast	[179]
	**Negative**	Breast	[122]
			[44]
		Ovary	[131]
			[142]
		Head and Neck	[180]
			[181]
		Melanoma	[174]
			[146]
	None	Melanoma	[128]

TA-DCs: tumor-associated dendritic cells; cDC2: conventional dendritic cell type 2; pDC: plasmacytoid dendritic cells.
